# Louis Pasteur

**Published:** 1895-11

**Authors:** 


### *EDITORIAL.*

The office of the Business Manager of THE JOURNAL is Nos. 311 and 312 Fitten building.

The editors are not responsible for views expressed by contributors.

Articles for publication should reach this office not later than the 15th instant.

Address all communications, and make all remittances payable to THE ATLANTA MEDICAL AND SURGICAL JOURNAL, Atlanta, Ga.

Reprints of articles will be furnished, when desired, at a nominal cost. Requests for the same should always be made *on the manuscript.*

### LOUIS PASTEUR.

In the death of Pasteur, which occurred September 28th, the scientific and medical worlds have sustained another great loss. Pasteur was more to France than Helmholtz to Germany or Huxley to England. He was educated for a chemist, and the first years of his active life were occupied with professional duties in the schools of Paris, Strassburg and Lille.

Among his first successful investigations, were his studies in crystallography, polarization of light, fermentation, the manufacture of wine, etc. He was the first to explode conclusively and finally the troublesome theory of "spontaneous generation" (1860). In 1865, at the request of the government, he undertook an investigation of the diseases of silkworms, which threatened to destroy the silk industries of France. After three years he discovered the cause (parasitic) and pointed out the remedy. He next began some studies in splenic fever, anthrax and chicken cholera, and these studies became to a large extent the foundation for the later science of bacteriology as well as for subsequent researches in serum therapy and immunization.

But it was especially in connection with hydrophobia that

Pasteur's name and work became so widely know. His studies on rabies began in 1880. Results were announced at the proper time and are too well known to require description. His theories on this subject were at first opposed by many good men, but are now generally accepted by scientists. Institutes for the treatment of this disease, based upon the principles which he enunciated, are now in successful operation in the most important cities, and named in his honor.

To show that republics are not always ungrateful, Pasteur had received almost every public honor which France could bestow, besides having enjoyed an annuity of $2,400 since 1874. He had also been the recipient of numerous foreign honors, medals and decorations. His life was a series of grand successes, which increased both our natural knowledge and material prosperity.

